# The stringent response promotes biofilm dispersal in *Pseudomonas putida*

**DOI:** 10.1038/s41598-017-18518-0

**Published:** 2017-12-22

**Authors:** Carlos Díaz-Salazar, Patricia Calero, Rocío Espinosa-Portero, Alicia Jiménez-Fernández, Lisa Wirebrand, María G. Velasco-Domínguez, Aroa López-Sánchez, Victoria Shingler, Fernando Govantes

**Affiliations:** 1 Centro Andaluz de Biología del Desarrollo, Universidad Pablo de Olavide/Consejo Superior de Investigaciones Científicas/Junta de Andalucía and Departamento de Biología Molecular e Ingeniería Bioquímica, Universidad Pablo de Olavide, Sevilla, Spain; 2 Department of Molecular Biology, Umeå University, Umeå, Sweden; 3 Present Address: Department of Microbiology and Immunology, Weill Cornell Medicine, New York, USA; 4 Present Address: Danmarks Tekniske Universitet. Novo Nordisk Foundation Center for Biosustainability. Kemitorvet. Bygning 220, 2800 Lyngby, Denmark

## Abstract

Biofilm dispersal is a genetically programmed response enabling bacterial cells to exit the biofilm in response to particular physiological or environmental conditions. In *Pseudomonas putida* biofilms, nutrient starvation triggers c-di-GMP hydrolysis by phosphodiesterase BifA, releasing inhibition of protease LapG by the c-di-GMP effector protein LapD, and resulting in proteolysis of the adhesin LapA and the subsequent release of biofilm cells. Here we demonstrate that the stringent response, a ubiquitous bacterial stress response, is accountable for relaying the nutrient stress signal to the biofilm dispersal machinery. Mutants lacking elements of the stringent response – (p)ppGpp sythetases [RelA and SpoT] and/or DksA – were defective in biofilm dispersal. Ectopic (p)ppGpp synthesis restored biofilm dispersal in a ∆*relA* ∆*spoT* mutant. *In vivo* gene expression analysis showed that (p)ppGpp positively regulates transcription of *bifA*, and negatively regulates transcription of *lapA* and the *lapBC*, and *lapE* operons, encoding a LapA-specific secretion system. Further *in vivo* and *in vitro* characterization revealed that the P*bifA* promoter is dependent on the flagellar σ factor FliA, and positively regulated by ppGpp and DksA. Our results indicate that the stringent response stimulates biofilm dispersal under nutrient limitation by coordinately promoting LapA proteolysis and preventing *de novo* LapA synthesis and secretion.

## Introduction

Alternation between a free-swimming planktonic lifestyle and the formation of structured highly cooperative polymer-encased sessile communities, also known as biofilms, is a staple of bacterial life in the environment^1^. Biofilm formation is a form of coordinated collective behaviour that has been regarded as an evolutionary precursor of developmental processes^2,3^. The biofilm developmental cycle proceeds through distinct stages of adhesion, proliferation and maturation, and is terminated by programmed biofilm dispersal^2–4^. Transition through these stages requires the timely production of different factors in response to environmental and physiological cues occurring during the biofilm developmental cycle, which involves a variety of signal transduction and regulatory pathways to connect such cues to the appropriate physiological responses^3^.

Unlike other means of biofilm escape, such as detachment or sloughing, biofilm dispersal is a genetically programmed response that enables bacterial cells to exit the biofilm in response to physiological or environmental conditions – i.e. conditions that are insufficient to provoke such effect in the absence of an active response by the cells^5^. Biofilm dispersal is generally preceded by a decrease in the intracellular concentration of cyclic diguanylate (c-di-GMP)^6–11^, a ubiquitous bacterial second messenger that mediates the transitions between the planktonic and sessile lifestyles, and is also involved in directing other diverse cellular processes, including motility, protein secretion, cell cycle progression and virulence^12–16^. Within a biofilm, the reduction of c-di-GMP levels stimulates the synthesis and release of hydrolytic enzymes to break down components of the extracellular polymeric substance (EPS) of the biofilm matrix^17^. As the hallmark of the transition from sessile to planktonic growth, decreased c-di-GMP levels lead to the cessation of the expression of biofilm-specific traits, such as synthesis of EPS components, and the induction of planktonic-specific traits, such as flagellar motility^5,12,13^. While factors involved in biofilm dispersal have been recognized in a handful of organisms^7,18–21^, the regulatory pathways connecting the environmental or physiological dispersal-inducing cues with such dispersal factors are for the most part unidentified.

Biofilms of the Gram-negative soil bacterium *Pseudomonas putida* undergo rapid dispersal in response to nutritional stress^22^. Studies performed in *P. putida* and the related species *P. fluorescens* have determined that dispersal is effected by proteolytic cleavage of the high molecular weight adhesin LapA by the periplasmic protease LapG. Dispersal conditions are signaled by a decrease in c-di-GMP concentration, which is sensed by the membrane-bound signal transduction protein LapD. LapD inhibits LapA proteolysis by sequestering LapG in the presence of high c-di-GMP levels, but inhibition is released when c-di-GMP levels are lowered, resulting in LapA cleavage and disassembly of cell-surface and cell-cell contacts^4,7,15,23^. We recently showed that the phosphodiesterase (PDE) BifA is responsible for the drop in c-di-GMP levels that signals biofilm dispersal in *P. putida* ^24^. Despite the detailed knowledge of the effector system for biofilm dispersal in this organism, the mechanisms that modulate the c-di-GMP levels in response to nutrient availability remain unknown.

The hyperphosphorylated guanine nucleotides guanosine 3′,5′-bis(diphosphate) (guanosine tetraphosphate or ppGpp) and guanosine 3′-diphosphate, 5′-triphosphate (guanosine pentaphosphate or pppGpp), collectively known as (p)ppGpp, are key players in the regulation of bacterial growth and metabolism^25^. In response to different conditions of nutritional or environmental stress, increased synthesis of (p)ppGpp results in major reprogramming of transcription, inhibition of DNA replication, and multiple changes in bacterial metabolism and behavior, a phenomenon known as the stringent response (SR)^26–28^. The SR is an adaptive physiological response aimed to increase survival under harsh conditions, and has also been shown to influence biofilm formation, antibiotic resistance, persistence and virulence^25,29^. In *P. putida* and other γ and β proteobacteria, (p)ppGpp is synthesized by two proteins: RelA, whose activity is induced under amino acid starvation, and SpoT, which responds to other forms of nutritional stress, such as carbon, iron, oxygen or fatty acid limitation, and also bears (p)ppGpp hydrolase activity^30^. In these organisms, the TraR-family protein DksA acts as an auxiliary factor of the SR by increasing the sensitivity of RNA polymerase (RNAP) to the cellular levels of (p)ppGpp, with the consequent synergistic inhibition or stimulation of transcription from kinetically sensitive promoters^28,31,32^.

In a genetic screen for *P. putida* factors involved in starvation-induced dispersal, we isolated two insertion mutants in *dksA* that showed a delayed biofilm dispersal phenotype^33^. Here we explore the role of the players of the stringent response, namely DksA and the (p)ppGpp synthetases RelA and SpoT, in the induction of biofilm dispersal in this organism.

## Results

### Stringent response mutants are defective in starvation-induced dispersal

**Figure 1:**
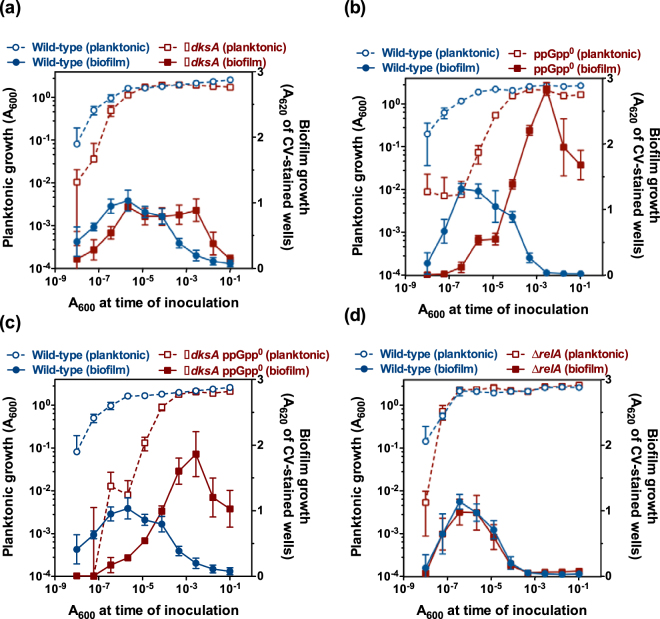
Dilution series-based planktonic and biofilm growth curves of stringent response mutants in LB. Planktonic (left axes, open symbols) or biofilm growth (right axes, closed symbols) is plotted against the initial A_600_ of each dilution. Blue circles represent the wild-type KT2440 strain and red squares represent the ∆*dksA* mutant MRB46 (**a**), the ppGpp^0^ mutant PP1922 (**b**), the ∆*dksA* ppGpp^0^ mutant MRB53 (**c**), or the ∆*relA* mutant PP1437 (**d**) Plates were incubated for 20 hours prior to measurement. Plots display one representative experiment of at least three biological replicates. Error bars represent the standard deviation of the six technical replicates.

We previously showed^33^ that a *P. putida* mutant bearing a transposon insertion in *dksA* showed a delayed dispersal response in biofilms formed on microtiter plate wells. *P. putida dksA* (*PP_4693*) is located upstream and in the same orientation to *PP_4694*, encoding glutamyl-Q tRNA(Asp) synthetase. Operon prediction by the DOOR algorythm^34^, as well as the lack of transcription start sites mapped by differential RNA sequencing upstream from *PP_4694* ^35^ strongly suggest that both genes are co-transcribed. Thus, to prevent polar effects on the downstream gene, we used allele replacement to construct MRB46, a derivative of the reference strain *P. putida* KT2440 bearing an unmarked in-frame deletion of *dksA*. In addition, we also generated the same ∆*dksA* mutation in PP1922, a KT2440 derivative unable to synthesize (p)ppGpp (henceforth designated ppGpp^0^), due to deletions of the (p)ppGpp sythetase-encoding genes *relA* and *spoT* ^32^. To analyse the ability of this mutant set to form and disperse biofilms in a microtiter dish setting, we performed dilution series-based growth curves, a method in which a dilution series is used to recapitulate the time-course of planktonic and biofilm growth^33^. To this end, the wild-type (KT2440), ∆*dksA* (MRB46), ∆*relA* (PP1437), ppGpp^0^ (PP1922) and ∆*dksA* ppGpp^0^ (MRB53) strains were serially diluted in LB, inoculated in microtiter plate wells and planktonic and biofilm growth was monitored after 20 hour incubation (Fig. 1).

Consistent with the behaviour of the insertion mutant, the ∆*dksA* strain showed somewhat slower planktonic and biofilm growth relative to the wild-type (Fig. 1a). In addition, this mutant failed to disperse the biofilm at the onset of stationary phase. Instead, biofilm biomass was retained until well into stationary phase and then a delayed dispersal response was observed. The ppGpp^0^ and ∆*dksA* ppGpp^0^ mutants exhibited similar behaviour: after a prolonged lag phase, planktonic and biofilm growth occurred similarly to that of the wild-type, but the biofilm was not dispersed at the onset of stationary phase (Fig. 1b,c). Rather, biofilm biomass accumulated to peak at levels 2- to 3-fold greater than those in the wild-type and, even though biofilm levels decreased somewhat during late stationary phase, a full dispersal response did not occur. Extended incubation of the ∆*dksA* ppGpp^0^ mutant dilution set to 26 hours resulted in a similar increase in biofilm accumulation, while dispersal was still not observed (Supplementary Fig. S1). These results strongly suggest that the lack of the components of the stringent response is associated with a permanent defect in biofilm dispersal. Interestingly, a ∆*relA* mutant (PP1437) showed a phenotype indistinguishable from that of the wild-type (Fig. 1d), suggesting that the (p)ppGpp synthesis activity of SpoT is sufficient to support a normal biofilm growth cycle under our experimental conditions.

A similar set of dilution-based growth curves was performed in K10T-1, a medium containing tryptone and glycerol that is commonly used for biofilm studies in *Pseudomonas fluorescens* ^36^ (Supplementary Fig. S2). The general pattern of planktonic growth and biofilm accumulation and dispersal was similar to that observed in LB, but the dispersal defect of the ∆*dksA* mutant was more severe, as biofilm biomass was not diminished in late stationary phase. Complementation assays confirmed that the lack of DksA is responsible for these phenotypes, as insertion of a miniTn7BB-Gm derivative expressing *dksA* from its own promoter rescued both the growth and biofilm dispersal defects of the ∆*dksA* strain (Supplementary Fig. S3). Taken together, our results strongly suggest that an active stringent response involving DksA and (p)ppGpp synthesis is required for starvation-induced biofilm dispersal.

**FIgure 2:**
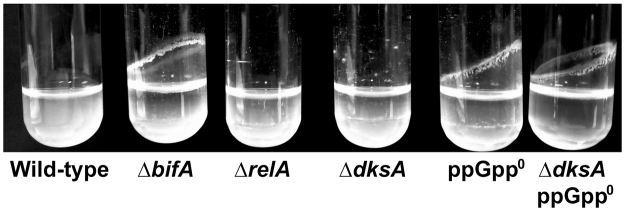
Pellicle formation by stringent response mutants. The picture shows a representative assay of three biological replicates.

The ability of our mutant set to form a biofilm at the medium-air interphase (pellicle) was also assessed using shaking culture tubes containing K10T-1 medium (Fig. 2). While the wild-type strain showed little or no pellicle biomass, the KT2442-derived ∆*bifA* strain MRB32 (used here as a positive control) displayed a thick pellicle, as previously described^23^. The ∆*relA* and ∆*dksA* mutants displayed behaviour similar to that of the wild-type, while the ppGpp^0^ and ∆*dksA* ppGpp^0^ mutants showed pellicle levels comparable to those in the ∆*bifA* mutant. The correlation between the inability to synthesize (p)ppGpp, high levels of biofilm and pellicle formation suggests that (p)ppGpp is a negative regulator of cell adhesion and biofilm formation.

### Ectopic (p)ppGpp synthesis restores biofilm dispersal in the ppGpp^0^ mutant

**Figure 3:**
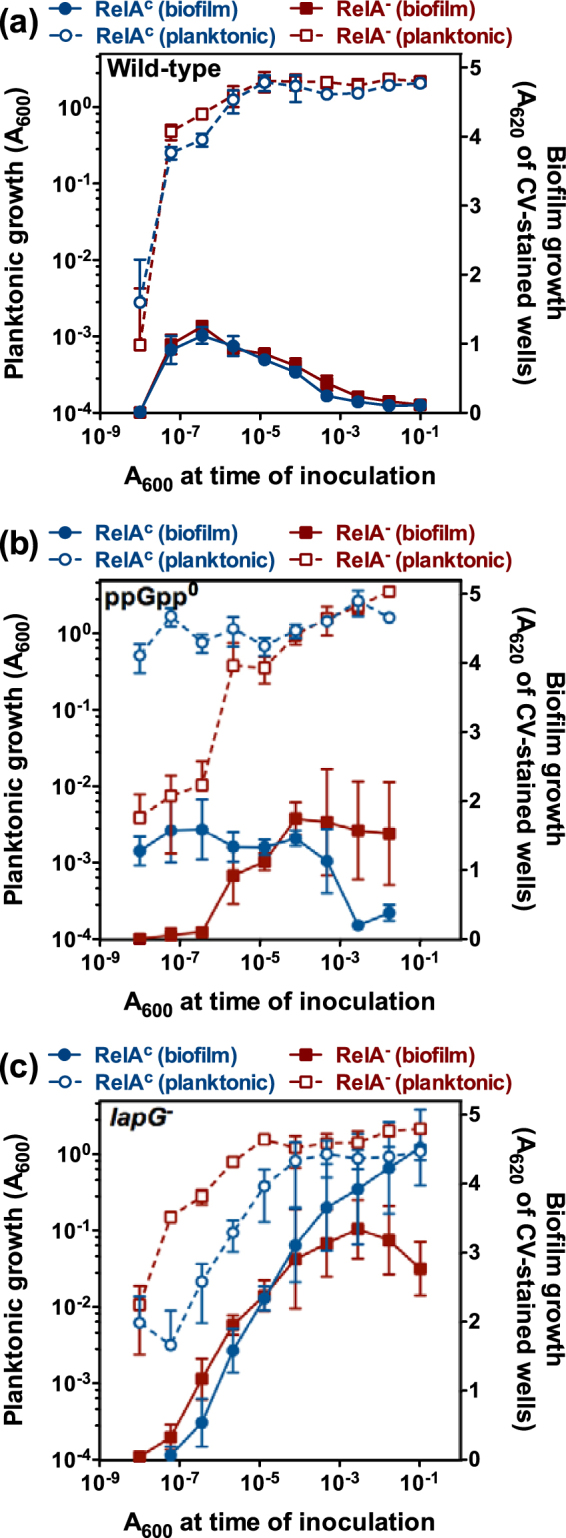
Dilution-based planktonic and biofilm growth curves of *P. putida* strains producing RelA derivatives. Planktonic (left axes, open symbols) or biofilm growth (right axes, closed symbols) is plotted against the initial A_600_ of each dilution. Panel (a): blue circles and red squares represent the wild-type strain KT2440 bearing the constitutive RelA^∆456–743^-producing plasmid pMRB160 and the inactive RelA^∆332–743^-producing plasmid pMRB162, respectively. Panel (b): blue circles and red squares represent the ppGpp^0^ strain PP1922 bearing the constitutive RelA^∆456–743^-producing plasmid pMRB160 and the inactive RelA^∆332–743^-producing plasmid pMRB162, respectively. Panels (c): blue circles and red squares represent the *lapG* ^−^ strain MRB1 bearing the constitutive RelA^∆456–743^-producing plasmid pMRB160 and the inactive RelA^∆332–743^-producing plasmid pMRB162, respectively. The growth medium was LB. Plates were incubated for 20 hours prior to measurement. Plots display one representative experiment of at least three biological replicates. Error bars represent the standard deviation of the six technical replicates.

To further characterize the role of (p)ppGpp in the biofilm growth cycle, we determined the effect of ectopic (p)ppGpp synthesis on planktonic and biofilm growth of the wild-type and ppGpp^0^ strains. To this end, plasmids pMRB160 and pMRB162 were constructed to produce the C-terminally truncated derivatives of the *Escherichia coli* RelA protein RelA^∆456–743^, displaying constitutive (p)ppGpp synthetic activity, and RelA^∆332–743^, which is inactive^37,38^, from the *nahR*-P*sal* expression system^39^. The expressing plasmids were transferred to the wild-type KT2440 and ppGpp^0^ PP1922 strains and biofilm and planktonic growth was assessed by means of serial dilution-based growth curves. Even though the P*sal* promoter is inducible by salicylate, induction of RelA^∆456–743^ synthesis was inhibitory to growth, and therefore assays were only performed in the absence of salicylate (Fig. 3).

The planktonic and biofilm growth curves of the wild-type strain bearing the RelA^∆456–743^ and RelA^∆332–743^-producing plasmids (Fig. 3a) were similar to that observed in the absence of plasmid (Fig. 1a–d). When tested in the ppGpp^0^ background, the inactive RelA^∆332–743^-producing strain also showed a behaviour similar to that of the plasmid-free ppGpp^0^ strain (Fig. 1b). In contrast, production of the constitutive RelA^∆456–743^ derivative rescued both the planktonic growth and biofilm dispersal defects associated to the lack of (p)ppGpp (Fig. 3b), suggesting that basal transcription from P*sal* provides sufficient amount of RelA^∆456–743^ to promote biofilm dispersal, while not being detrimental to growth. It could be argued that ectopic (p)ppGpp production may promote non-specific biofilm detachment by mechanisms unrelated to the starvation-induced biofilm dispersal signalling pathway. To test this hypothesis, the effect of RelA^∆456–743^ and RelA^∆332–743^ production was also tested in pMRB160 and pMRB162-bearing *P. putida* strain MRB1^33^. This mutant lacks the periplasmic protease LapG, the effector element of starvation-induced dispersal response, required for LapA cleavage. Although production of the constitutive RelA^∆456–743^ derivative provoked a considerable delay in planktonic growth of MRB1 (Fig. 3c), both plasmid-bearing strains displayed high levels of biofilm that failed to reproduce the dispersal response upon entry in stationary phase, much like our previous observations with plasmid-free MRB1^33^. These results are consistent with the hypothesis that (p)ppGpp induces a dispersal response functionally similar to the previously characterized starvation-induced dispersal response.

### The flagellar σ factor FliA and the stringent response coordinately regulate *bifA* transcription

**Figure 4:**
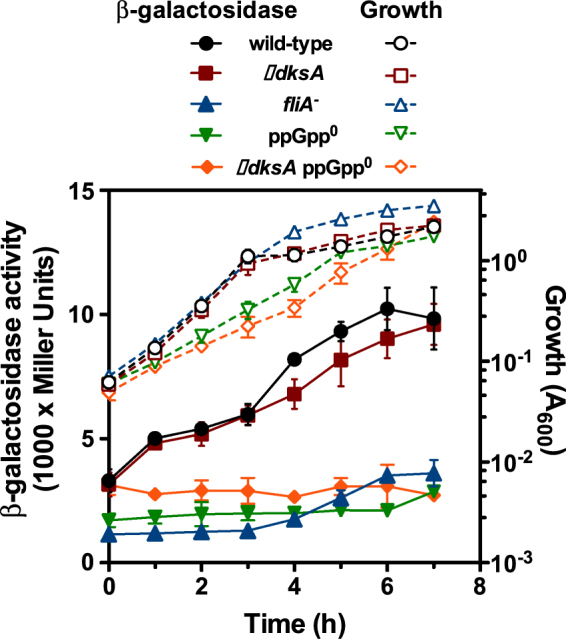
*In vivo* expression of the P*bifA* promoter. Growth (closed symbols, right Y axis) and β-galactosidase activity (open symbols, left Y axis) from wild-type (KT2440; black circles), ∆*dksA* (MRB46; red squares), *fliA* (KT2440 *fliA*::*aphA-3*; blue triangles), ppGpp^0^ (PP1922; green inverted triangles) and ∆*dksA* ppGpp^0^ (MRB53; orange diamonds) *P. putida* strains bearing the P*bifA-gfp*mut3-*lacZ* fusion transcriptional reporter plasmid pMRB68. The plot represents the averages and standard deviations of three independent assays performed in duplicate.

Our recent work revealed that BifA is the PDE responsible for c-di-GMP depletion during biofilm dispersal. On the other hand, our results above establish that presence of the elements of the SR is required for biofilm dispersal. In *P. putida*, *bifA* transcription is dependent on the flagellar σ-factor FliA^40^. In addition, it has been shown that *P. putida* RNAP utilizing FliA is co-ordinately regulated by DksA and (p)ppGpp to elevate the expression of the *aer-2* gene, encoding an oxygen-and metabolism-sensor protein^41^. Therefore, we reasoned that, similarly to *aer-2*, *bifA* transcription may be subjected to dual regulation by FliA and the SR. To test this possibility, plasmid pMRB68, harbouring a P*bifA-gfp-lacZ* fusion was transferred to KT2440 and its SR and *fliA* ^−^ mutant derivatives and expression was monitored across the growth curve by means of β-galactosidase assays (Fig. 4).

Expression of the P*bifA* promoter in the wild-type strain increased 3-fold as the culture progressed from exponential to stationary phase. A similar behaviour was observed in the ∆*dksA* mutant, suggesting that DksA does not greatly influence *bifA* expression in these conditions. In contrast, low, basal expression levels were observed in the *fliA* ^−^, ppGpp^0^ and ∆*dksA* ppGpp^0^ mutants. These results confirm the FliA-dependence of P*bifA* transcription and indicate that (p)ppGpp synthesis is required for high level expression of *bifA*.

To determine whether the effect of FliA and the SR on *bifA* is exerted at the level of P*bifA* transcription initiation, single-round *in vitro* transcription assays were performed. These assays used plasmid pVI2407, containing the P*bifA* promoter, as a template, purified *P. putida* core RNA polymerase, FliA, and DksA, and ppGpp. As a control, similar reactions were performed using plasmid pVI1011, which bears the previously analysed FliA-, DksA- and ppGpp-regulated *P. putida* P*aer2* promoter^41^.

**Figure 5:**
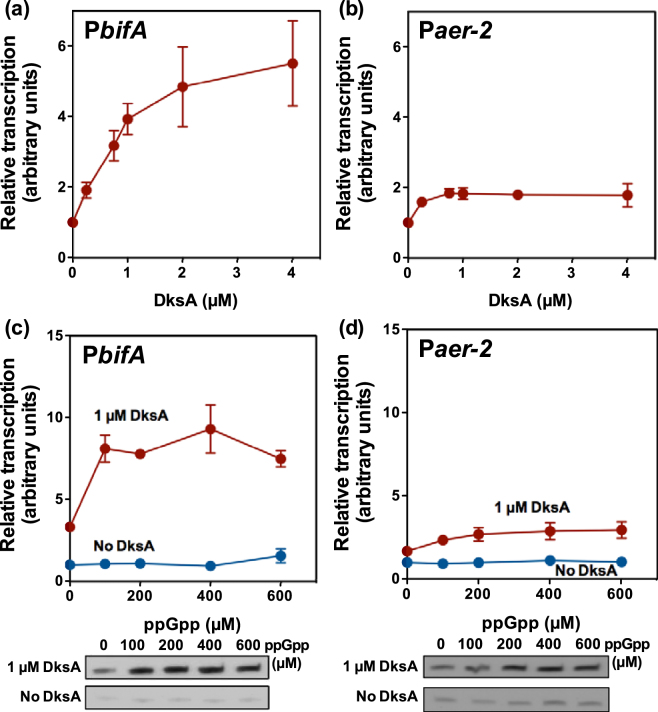
*In vitro* transcription of the P*bifA* and P*aer-2* promoters. Panels (a) and (b): DksA titration on the P*bifA* (**a**) and P*aer-2* (**b**) promoters. Panels (c) and (d): ppGpp titration on the P*bifA* (**c**) and P*aer-2* (**d**) promoters, in the absence (blue circles) and in the presence (red circles) of 1 µM DksA. Transcript levels are expressed relative to the amount obtained in the absence of ppGpp and DksA, which is arbitrarily set to 1. The plots represent the averages and standard deviations of three independent assays. Panels (c) and (d) include images of representative gels for each assay.

In the presence of FliA-RNAP but in the absence of ppGpp and DksA the levels of P*bifA* transcript were low. Transcript levels were increased in the presence of DksA, to reach a maximum 6-fold stimulation at 4 µM DksA (Fig. 5a). While ppGpp did not substantially affect P*bifA* transcription in the absence of DksA (Figs 5c and S4a), simultaneous addition of ppGpp and DksA resulted in the synergistic stimulation of P*bifA*: while 1 µM DksA alone provoked a 3-fold increase in transcription relative to the condition with no protein or effector added, stimulation was increased up to 5- to 9-fold when 1 µM DksA was combined with 100, 200, 400 or 600 µM ppGpp (Figs 5c and S4a). Conversely, 100–600 µM ppGpp did not stimulate transcription noticeably in the absence of DksA, but provoked a 2- to 3-fold increase in the transcript levels in the presence of 1 µM DksA relative to those obtained with 1 µM DksA in the absence of ppGpp. Consistent with previous results^41^, only a comparatively modest regulation of the P*aer2* promoter, amounting to a maximum 2-fold stimulation by DksA alone (Fig. 5b) and a 3-fold combined effect of 1 µM DksA plus 200–600 µM ppGpp (Figs 5d and S4b), was observed. Taken together these results confirm that the P*bifA* promoter is FliA-dependent and performance of FliA-RNAP at this promoter is directly stimulated by ppGpp and DksA *in vitro*. Furthermore, the high extent of the stimulation of the P*bifA* promoter indicates that the global effectors of the SR – ppGpp and DksA – can strongly influence transcription by FliA-RNAP.

### (p)ppGpp negatively regulates LapA synthesis and secretion

**Figure 6:**
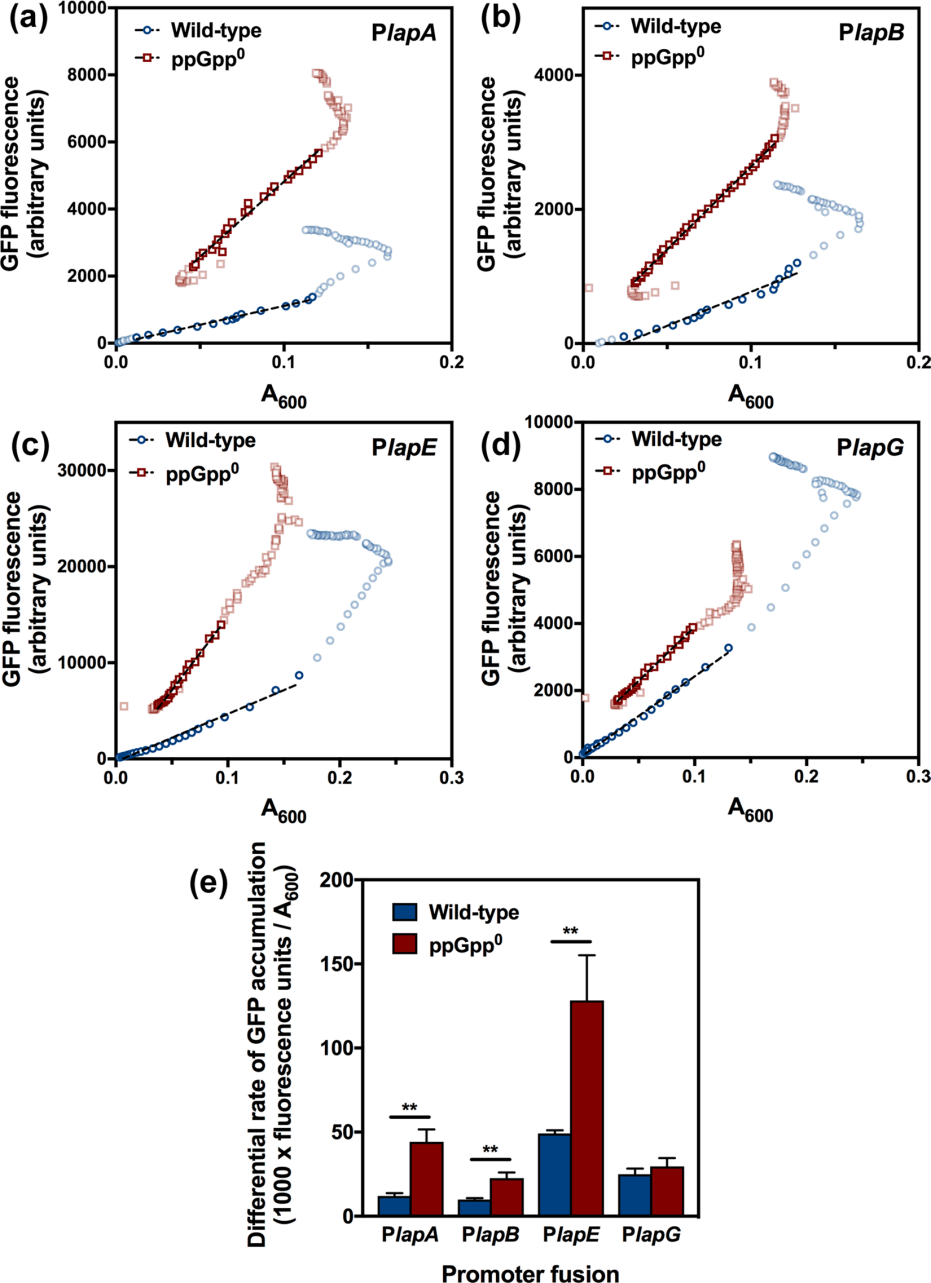
Effect of (p)ppGpp on the expression of biofilm dispersal-related genes. (**a**–**d**). Fluorescence *vs*. absorbance plots of the data collected from one representative replicate of the assays performed with the P*lapA* (**a**), P*lapBC* (**b**), P*lapE* (**c**) and P*lapGD* (**d**) promoter fusions. Blue circles denote the wild-type strain KT2440 and red squares denote de ppGpp^0^ strain PP1922. Highlighted data points were used for linear regression, which is shown as a hatched black line. R-squared values were always greater than 0.98. (**e**). Differential rates of GFP accumulation. Columns and error bars represent the averages and standard deviations obtained from from at least three independent biological replicates. Stars denote statistical significance assessed by two-tailed T-tests not assuming equal variance (*p < 0.05; **p < 0.01; ***p < 0.001).

In addition to BifA, other elements involved in the dispersal response are LapD, a c-di-GMP sensor that regulates the proteolytic activity of the LapG, and LapA, a high molecular weight outer membrane adhesin. On the other hand, LapA localization at the outer membrane is strictly dependent on a ABC-type secretion system, comprised of LapB, LapC and LapE^42^. In order to identify possible additional biofilm dispersal-related targets of SR response signaling, plasmids pMRB67, pMRB66, pMRB240 and pMRB241, bearing transcriptional *gfp-lacZ* fusions to the P*lapA* and P*lapBC*, P*lapE*, and P*lapGD* promoters, respectively, were introduced into the wild-type KT2440 and the ppGpp^0^ mutant PP1922, and expression was assessed by online monitoring of GFP fluorescence during the growth curve. Differential rates of GFP fluorescence accumulation during exponential phase were calculated from the slopes of the fluorescence *vs*. absorbance plots (Fig. 6 a–d). Expression from the P*lapA*, P*lapBC* and P*lapE* promoters was increased 4-, 2- and 3-fold respectively in the ppGpp^0^ relative to the wild-type strain, while, P*lapGD* expression was not significantly altered in these conditions (Fig. 6 e). These results indicate that, in addition to stimulating the synthesis of the c-di-GMP-depleting PDE BifA, (p)ppGpp acts directly or indirectly by diminishing the synthesis and secretion of the high molecular weight biofilm adhesin LapA.

## Discussion

Programmed biofilm dispersal is a mechanism described in multiple bacteria that enables them to actively exit the biofilm structure and resume a planktonic lifestyle in response to environmental and/or physiological cues. Here we describe the involvement of the components of the SR, the nucleotide alarmone (p)ppGpp and the protein DksA, in the induction of biofilm dispersal in the Gram-negative soil bacterium *P. putida* KT2440. The SR and (p)ppGpp have previously shown to influence biofilm development in multiple bacterial species, including *Listeria monocytogenes*, *Streptococcus mutans*, *Enterococcus faecalis*, *Vibrio cholerae* and uropathogenic *E. coli* ^29,43–47^. In contrast to our findings, induction of the SR stimulated biofilm formation in these organisms, and this positive effect has been used as an argument for the development of SR inhibitors as therapeutic agents against biofilm infections^48–50^. However, our results indicate that this is not always the case and, depending on the bacterial species, the result of treatment with SR antagonists may be exactly the opposite – i.e., stimulation of biofilm formation. Interestingly, (p)ppGpp is a negative regulator of biofilm growth in *Francisella novicida* ^51,52^, although the mechanisms involved have not been explored further.

Our phenotypic analysis of ∆*dksA*, ppGpp^0^ and ∆*dksA* ppGpp^0^ strains revealed defects in starvation-induced biofilm dispersal. Strains unable to synthesize (p)ppGpp (ppGpp^0^ and ∆*dksA* ppGpp^0^) were strongly impaired in biofilm dispersal, at least within the 20–26 h time frame of our experiments (Fig. 1; Supplementary Figs S1 and S2), and displayed elevated biofilm levels, both in a microtiter plate submerged biofilm model (Fig. 1; Supplementary Figs S1 and S2), and in a medium-air interphase (Fig. 2) setting. Similarly elevated biofilm levels have also been observed in other *P. putida* mutants lacking elements involved in biofilm dispersal, such as LapG^7,33^, or BifA^24,33^. In contrast to the (p)ppGpp^0^ strain, a wild-type dispersal response was observed in the ∆*relA* mutant (Fig. 1), indicating that SpoT-dependent (p)ppGpp synthesis is sufficient to trigger dispersal under our culture conditions. An analogous situation has been reported for *Salmonella* virulence in mice, which is attenuated in a ppGpp^0^, but not in a ∆*relA* mutant^53–55^. On the other hand, the defects observed in the ∆*dksA* strain were comparatively small relative to those observed in the ppGpp^0^ and ∆*dksA* ppGpp^0^ strains – biofilm dispersal was delayed but not abolished, and biofilm overgrowth was not observed (Figs 1 and 2; Supplementary Figs S1 and S2). This relatively mild *in vivo* phenotype, and the fact that *dksA* inactivation did not have an additive effect in a ppGpp^0^ background may be attributed to only a partial dependence of the dispersal response on DksA, or alternatively, to the presence of redundant functions that may partially substitute for DksA in its absence (also see below).

We traced the effect of the stringent response on the starvation-induced dispersal response to the transcriptional control of *bifA*, which encodes a c-di-GMP phosphodiesterase responsible for the drop in c-di-GMP concentration that triggers biofilm dispersal in *P. putida* ^24^, and *lapA*, *lapBC* and *lapE*, encoding the high molecular weight adhesin and the LapA-specific secretion system^42^. During revision of the present manuscript, a paper showing the involvement of (p)ppGpp in the regulation of biofilm formation in *P. putida* KT2440 was published^56^. These authors identified biofilm-related targets for positive (*lapA* and the exopolysaccharide synthesis clusters *peb* and *bcs*) and negative regulation (*lapF*, encoding a second adhesin and the exopolysaccharide synthesis cluster *pea*). These results are complementary to our own observations, and reinforce the notion that the stringent response is an integral component of the regulatory network of the biofilm cycle, acting directly or indirectly on multiple factors.

Further characterization of the P*bifA* promoter revealed that *bifA* transcription is subjected to dual regulation *in vivo*, by the flagellar σ factor FliA, as previously described^40^, and by (p)ppGpp (Fig. 4). Furthermore, single-round *in vitro* transcription showed that the P*bifA* promoter is regulated synergistically by ppGpp and DksA (Fig. 5), thus substantiating the notion that *bifA* is a target for activation through the SR. Despite the clear *in vitro* evidence for the involvement of DksA in stimulation of FliA-dependent transcription from P*bifA*, the effect of *dksA* inactivation on P*bifA* stimulation *in vivo* was negligible (Fig. 4). We can envisage two plausible explanations for this apparent contradiction between the *in vivo* and *in vitro* data, both of which involve functional replacement by another family member. Firstly, the *P. putida* KT2440 genome contains two additional *dksA* paralogs: *PP_2220*, which encodes a protein with 68% similarity and 45% identity to *P. aeruginosa* DksA2, and *PP_3037*. Given that in *P. aeruginosa*, DksA2 is able to functionally replace DksA^57^, it is possible that the *P. putida* PP2220 and/or PP3037 proteins may simply take over the role of DksA. Secondly, DksA belongs to a family bacterial transcription factors that includes the GreA and GreB proteins amongst others, all of which bind and penetrate the secondary channel of RNAP to access the active site. In *E. coli*, partial redundancy and competition between GreA, GreB and DksA has been thoroughly documented and interplay between these factors can lead to discrepancies between *in vivo* and *in vitro* effects^44,58–60^. Solving which of these alternative possibilities is the case for P*bifA* is the subject of ongoing investigations.

**Figure 7:**
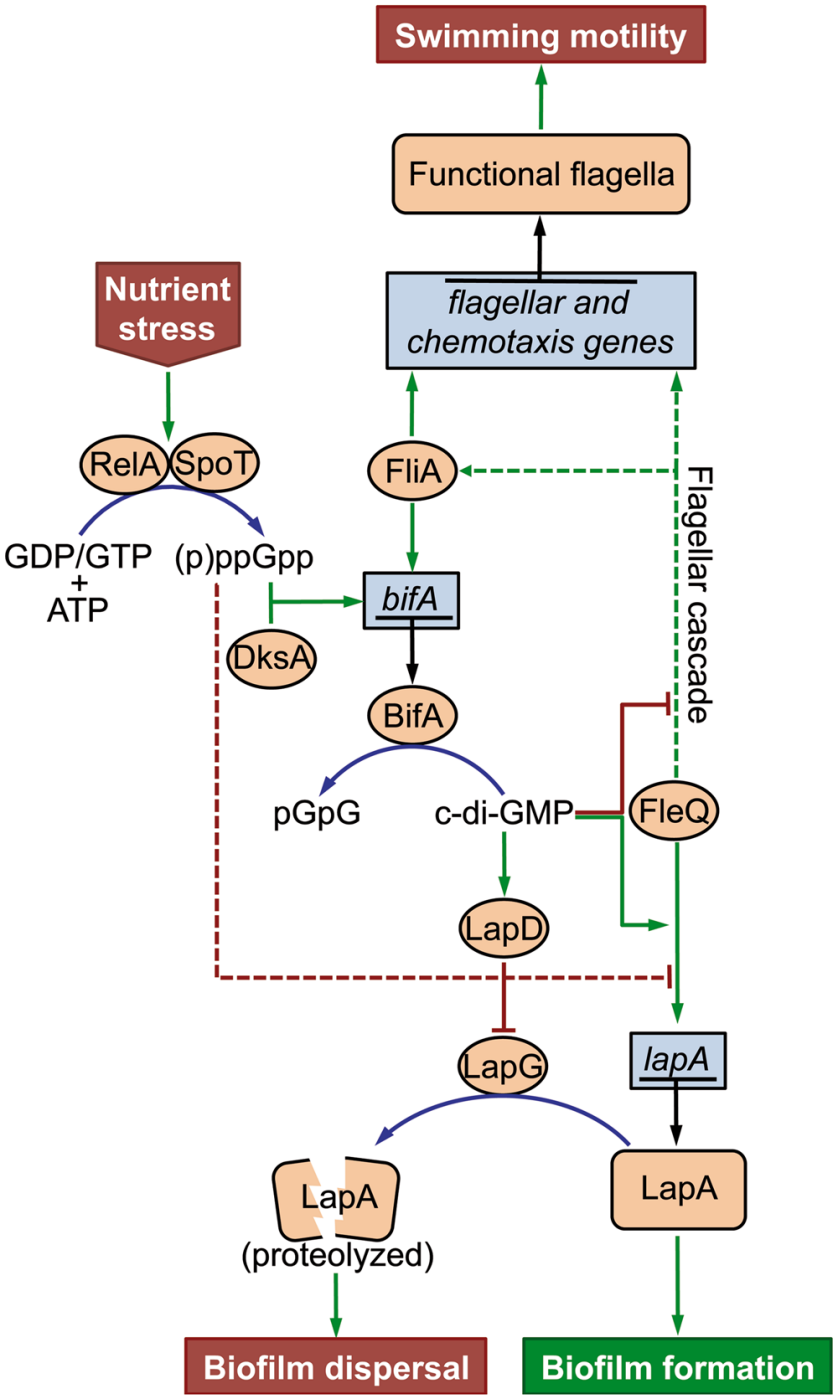
Working regulatory model. Schematic of a regulatory circuit for starvation-induced biofilm dispersal in *P. putida*. Genes are surrounded by blue boxes. Proteins are surrounded by orange ovals or rounded boxes. Signaling molecules and their precursors and degradation products are not boxed or encircled. Green arrows denote positive effects. Red T-shaped line ends denote negative effects. Blue lines denote the effect of enzyme catalysis and black lines denote the effect of gene expression. Hatched lines indicate indirect/complex effects. Red shapes denote the input (nutrient stress) and output (biofilm dispersal and resumption of swimming motility) of the starvation-induced dispersal circuit. A green shape denotes biofilm formation, occurring when the starvation-induced dispersal circuit is inactive.

Based on the results presented here and previous work by others and us, we propose a regulatory circuit for starvation-induced biofilm dispersal in *P. putida* as depicted in Fig. 7. This circuit connects nutrient stress with its ultimate outcome, namely LapA proteolysis that allows the bacterium to exit the biofilm and resume a planktonic lifestyle. In this model, nutrient limitation is signaled via the stringent response RelA and/or SpoT (p)ppGpp synthetases. Although we do not currently know the identity of the signal, our results indicate that SpoT, but not RelA, is required for this response; nevertheless, RelA may also be involved under a different growth regimes. The resulting elevated (p)ppGpp levels, along with the auxiliary protein DksA and the flagellar σ factor FliA then lead to stimulation of *bifA* transcription and hence increased levels of the phosphodiesterase BifA, which is responsible for depleting the c-di-GMP pool prior to biofilm dispersal^24^. The subsequent decrease in c-di-GMP levels would predictably release LapD inhibition of LapG protease activity, resulting in cleavage of LapA from the cell surface and biofilm dispersal^7,22^. We recently demonstrated that c-di-GMP inhibits FleQ-dependent activation of the flagellar cascade^61^, while stimulating FleQ-regulated synthesis of the biofilm matrix components LapA and cellulose^61,62^. Given those findings, we consider it likely that BifA-dependent c-di-GMP depletion would also result in concurrent cessation of LapA and cellulose synthesis accompanied by the stimulation of flagellar synthesis. In turn, FliA activation during flagellar assembly would probably increase BifA synthesis further, thus completing a positive feedback-loop to tightly couple biofilm dispersal with the *de novo* synthesis of the flagellar apparatus in preparation for the resumption of a planktonic lifestyle.

The model outlined above places BifA as a central integrator of the nutrient stress and biofilm dispersal responses mediated through the global regulatory networks of (p)ppGpp and c-di-GMP. In addition, we have recently shown that FleQ and FliA influence the synthesis of several other enzymes involved in c-di-GMP metabolism in *P. putida* ^61^. Hence, it appears likely that a complex sub-network of interactions could also act to additionally fine-tune c-di-GMP levels as required for the physiological regulation of the switch between the planktonic and sessile lifestyles and vice versa.

## Methods

### Bacterial strains and growth conditions

Bacterial strains used in this work are summarized in Supplementary Table 1. Planktonic cultures of *E. coli* and *P. putida* strains were routinely grown in Luria-Bertani (LB) broth^63^ at 37 °C and 30 °C, respectively, with 180 rpm shaking. For online fluorescence monitoring, 1/10 strenght LB was used to minimize background fluorescence. For solid media, Bacto-Agar (Difco) was added to a final concentration of 18 g l^−1^. Antibiotics and other additions were used, when required, at the following concentrations: ampicillin (100 mg l^−1^), carbenicillin (0.5 g l^−1^), kanamycin (25 mg l^−1^), rifampicin (10 mg l^−1^), chloramphenicol (15 mg l^−1^), gentamycin (10 mg l^−1^), tetracycline (5 mg l^−1^), 5-bromo-4-chloro-3-indoyl-β-D-galactopyranoside (X-gal) (25 mg l^−1^) and sodium salicylate (2 mM). All reagents were purchased from Sigma-Aldrich, except for X- Gal, which was purchased from Fermentas.

### Plasmid and strain construction

Plasmids and oligonucleotides used in this work are summarized in Supplementary Table 1. All DNA manipulations were performed following standard procedures^63^. Restriction and modification enzymes were used according to the manufacturers instructions (Fermentas, Roche and NEB). When required, blunt ends were generated using the Klenow fragment or T4 DNA polymerase. *E. coli* DH5α was used as a host in cloning procedures. All cloning steps involving PCR were verified by commercial sequencing (Secugen). Plasmid DNA was transferred to *E. coli* and *P. putida* strains by transformation^64^, triparental mating^65^ or electroporation^66^. Site-specific integration of miniTn7 derivatives in *P. putida* strains was performed essentially as described^67^.

To construct *P. putida* strains with an in-frame deletion of the *dksA* gene, 701 bp and 780 bp from the upstream and downstream chromosomal regions flanking *dksA* were PCR-amplified with oligonucleotide pairs dksAFupstm/dksARupstm (upstream region) and dksAFdwstm/dksARdwstm (downstream region). The PCR products were cleaved with EcoRI and BamHI or BamHI and HindIII, respectively, and three-way ligated into EcoRI- and HindIII-digested pEX18Tc, yielding pMRB33. A FRT-flanked kanamycin resistance cassette was generated by cloning the BamHI-excised kanamycin resistance gene from pUTminiTn5-Km into EcoRV-digested pPS854 to generate plasmid pMPO284. The BamHI-excised FRT-flanked kanamycin resistance cassette was then cloned into the BamHI site in pMRB33, yielding pMRB38. This plasmid was transferred to *P. putida* KT2440 by electroporation. Selection of integration, allelic replacement and FLP-mediated excision of the kanamycin resistance marker was performed essentially as described^68,69^. The structure of the deleted *dksA* locus was verified by PCR and Southern blot. For construction of a ∆*dksA* derivative of the ppGpp^0^ strain PP1922, the kanamycin resistance cassette in pMRB38 was replaced by a streptomycin/spectinomycin resistance cassette from pUTminiTn5-Sm/Sp, which was first excised with BamHI and cloned at the EcoRV site between the FRT sites of pPS854 to yield pMRB97. The FRT-flanked Str^r^/Spec^r^ cassette was then excised with BamHI and cloned into BamHI-linearized pMRB33 to produce pMRB98, which was subsequently used to generate the *dksA* deletion in PP1922 as described above for pMRB33 in KT2440. *P. putida* strains with deletions within *relA* and *spoT* were generated as previously described^32^ using suicide plasmids pVI681 and pVI682, and double site recombination, with a KT2440 derivative harbouring miniTn5-Tel as the recipient.

The truncated versions of *E. coli relA*, encoding RelA^∆456–743^ and RelA^∆332–743^ were excised from pALS13 and pALS14, respectively, by EcoRI and HindIII digestion and cloned into pBBR1MCS-4 digested with the same enzymes to yield pMRB153 and pMRB154. Subsequently, the 1075 bp *nahR*-P*sal* expression cassette was obtained from XbaI- and SpeI-digested pMRB120 and cloned into the single XbaI site in pMRB153 and pMRB154 in the same orientation as the *relA* derivatives to yield pMRB160 and pMRB162.

PCR fragments containing the P*lapE* and P*lapGD* promoter regions were inserted in the directional TOPO^®^ cloning vector pENTR^TM^/D-TOPO^®^ and subsequently transferred using the Gateway® recombination technology (Thermo Fisher Scientific) into the Gateway^®^ *gfp*mut3-*lacZ* fusion vector pMRB3, to produce pMRB240 and pMRB241, as previously described^61^. A 217 bp synthetic DNA fragment (GenScript) containing the putative P*bifA* promoter region (positions −198 to +3) and flanked by EcoRI and BamHI sites was digested with these enzymes and cloned into EcoRI- and BamHI-cleaved pTE103 to yield the P*bifA in vitro* transcription template plasmid pVI2407.

### Biofilm growth and quantification

For most procedures involving biofilm growth, overnight cultures grown in LB or K10T-1 broth^34^ were diluted in the same medium to an A_600_ of 0.1 and 150 μl were dispensed into wells of Costar 96 microtiter polystyrene plates (Corning). The plates were incubated at 25 °C with moderate shaking (150 rpm) for the desired period of time and processed for planktonic and biofilm growth quantification, essentially as described^70^. Serial dilution-based growth curves were performed as described^33^. For each experiment, at least 3 biological replicates were assayed in sextuplicate.

For biofilm growth at the medium-air interphase (pellicle), fresh colonies were inoculated in glass tubes containing 5 ml of K10T-1 broth. Cultures were incubated overnight at 30 °C with shaking, after which the tubes were placed on a rack for 10 minutes and documented by digital photography.

### *In vivo* gene expression assays

β-galactosidase assays were used to examine the expression of the P*bifA* promoter in *P. putida* KT2440 and derivatives. Pre-inocula of bacterial strains harbouring the fusion plasmid pMRB67 were grown to the stationary phase in LB with carbenicillin. Cultures were diluted 100-fold in the same medium and incubated in the same conditions. Samples were withdrawn from the cultures at 1-hour intervals and β-galactosidase activity was determined from SDS- and chloroform-permeabilized cells as previously described^71^.

Alternatively, the rates of GFP accumulation during exponential growth were used to measure gene expression in cultures bearing *gfp-lacZ* fusions to different promoters. Overnight LB cultures of the fusion-bearing strains were diluted in 1/10 strength LB broth, and dispensed into the wells of a Costar 96 microtiter polystyrene plate (Corning). The plate was incubated in a Spark microtiter plate reader/incubator at 30 °C with 510 rpm shaking to mid-exponential phase. Cultures were then diluted in the same medium and incubated in the same conditions for 23 hours. A_600_ and GFP fluorescence (485 nm excitation, 535 nm emission) were monitored in 15-minute intervals during incubation. Differential rates of GFP fluorescence accumulation were calculated as the slopes of the linear regression (R^2^ > 0.98) performed on the exponential growth fluorescence *vs*. absorbance plots (Fig. 6).

### *In vitro* transcription assays

Nucleotides and [α-^32^P]-UTP were purchased from Roche Molecular Biochemicals and Perkin Elmer, respectively, while ppGpp was synthesized and purified as previously described^72^. Purification of *P. putida*-derived native core RNA polymerase, N-terminally His-tagged DksA (His-DksA) and C-terminally His-tagged FliA (FliA-His) was as previously described^41,73,74^. Protein concentration was determined using a BSA™ Protein Assay Kit (Pierce) with bovine serum albumin (BSA) as a standard.

Transcription assays were performed at 30 °C essentially as described previously^40^, using 10 nM supercoiled pTE103-based plasmids bearing the P*bifA* promoter (pVI2407) or the P*aer2* promoter (pVI1011) as the DNA template. Assays of a final volume of 20 µl were performed in T-buffer (35 mM Tris-acetic acid, pH 7.9; 70 mM potassium acetate; 5 mM magnessium acetate; 20 mM ammonium acetate; 1 mM dithiothreitol and 0.275 mg ml^−1^ BSA). Core RNA polymerase (10 nM) and FliA-His (40 nM) were pre-mixed and incubated for at least 5 min to allow holoenzyme formation. When required, ppGpp, His-DksA and/or His-DksA storage buffer were added to holoenzyme mixes and incubated for 5 min prior to addition of template DNA. Reactions were incubated for 20 minutes to allow open-complex formation. Single-round transcription was initiated in the presence of anti-RNase (Ambion) by the addition of NTPs (final concentration: ATP, 500 µM; GTP and CTP, 200 µM each; UTP, 80 µM and [α-^32^P]-UTP, 5 mCi at >3000 Ci mmol^−1^) and heparin (0.1 mg ml^−1^) to prevent re-initiation. After a further 10 minutes at 30 °C, the reactions were terminated by adding 5 µl of a stop/load mix [150 mM EDTA, 1 M NaCl, 14 M urea, 3% glycerol, 0.075% (w/v) xylene cyanol, 0.075% (w/v) bromophenol blue]. Transcripts were analysed on 7 M urea/5% (w/v) polyacrylamide sequencing gels and quantified using a Molecular Dynamics Phosphorimager.

### Data availability statement

All data generated or analyzed during this study are included in this published article (and its Supplementary Information files).

## Electronic supplementary material

Supplementary Information
