# Supplementary material for: The stringent response promotes biofilm dispersal in Pseudomonas putida
Source: Sci Rep. 2017 Dec 22;7:18055. doi: 10.1038/s41598-017-18518-0 (PMC5741744; doi:10.1038/s41598-017-18518-0)
Supplement: Supplementary file 1 — Supplementary Information [file 41598_2017_18518_MOESM1_ESM.doc]

**SUPPLEMENTARY INFORMATION**

**The stringent response promotes biofilm dispersal in *Pseudomonas putida***

Carlos Díaz-Salazar, Patricia Calero, Rocío Espinosa-Portero, Alicia Jiménez-Fernández, Lisa Wirebrand, María G. Velasco-Domínguez, Aroa López-Sánchez, Victoria Shingler and Fernando Govantes

**FIGURES**

**Supplementary Figure S1. Long-term dilution series-based growth curves of the ∆*dksA* ppGpp^0^ mutant.** Planktonic (left axis, open symbols) or biofilm growth (right axis, closed symbols) is plotted against the initial A_600_ of each dilution. Blue circles represent the wild-type KT2440 strain and red squares represent the ∆*dksA* ppGpp^0^ mutant MRB53Plates were incubated for 26 hours prior to measurement. The plot displays one representative experiment of three biological replicates. Error bars represent the standard deviation of the six technical replicates.


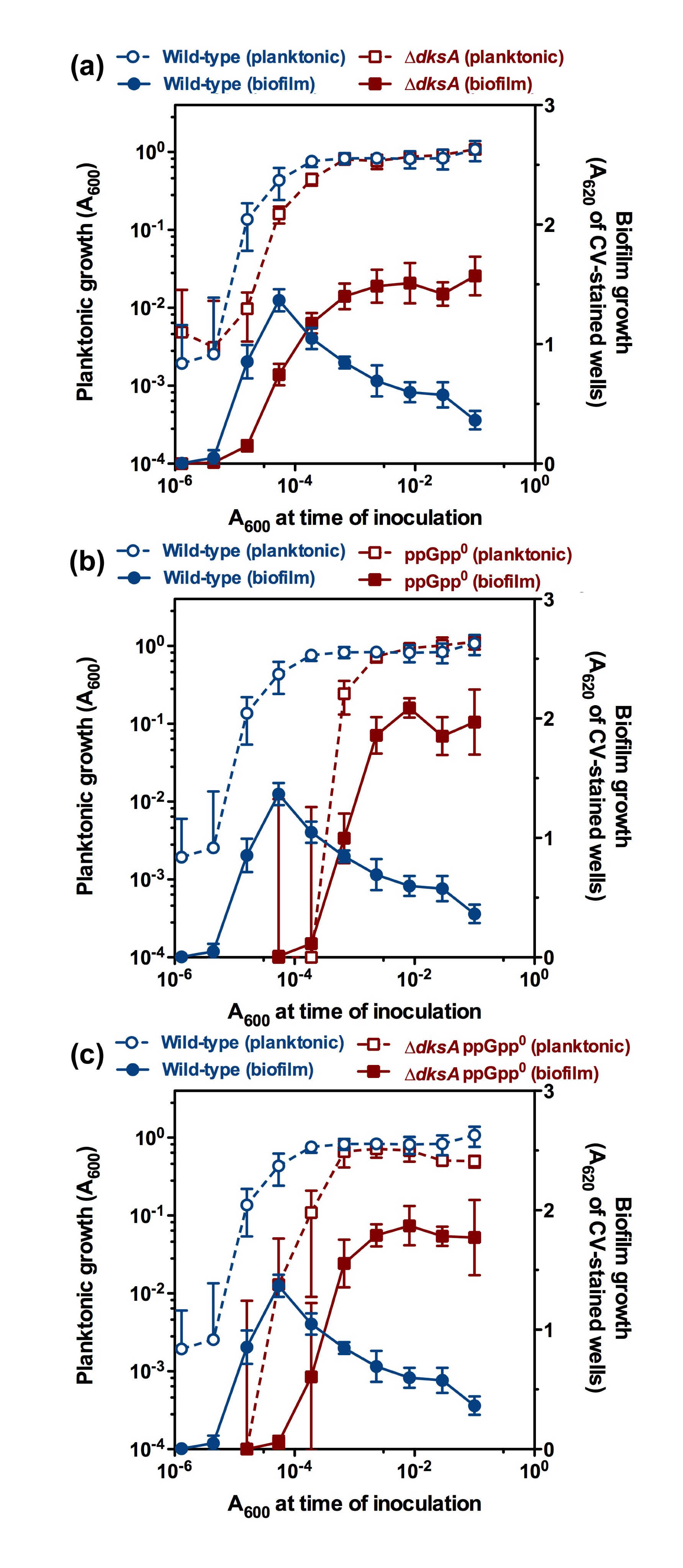


**Supplementary Figure S2. Dilution series-based planktonic and biofilm growth curves of stringent response mutants in K10T-1 medium.** Planktonic (left axes, open symbols) or biofilm growth (right axes, closed symbols) is plotted against the initial A_600_ of each dilution. Blue circles represent the wild-type KT2440 strain and red squares represent the ∆*dksA* mutant MRB46 (**A**), the ppGpp^0^ mutant PP1922 (**B**), or the ∆*dksA* ppGpp^0^ mutant MRB53 (**C** Plates were incubated for 24 hours prior to measurement. Plots display one representative experiment of at least three biological replicates. Error bars represent the standard deviation of the six technical replicates.

**Supplementary Figure S3. Complementation analysis of the ∆*dksA* mutant in K10T-1 medium.** Quantification of planktonic (**a**) and biofilm growth (**b**) from KT2440 bearing the empty vector miniTn*7*BB-Gm (wt/Ø), KT2440 bearing the *dksA*-expressing miniTn*7*BB-Gm[P*dksA-dksA*] (wt/*dksA*), MRB46 bearing the empty vector miniTn*7*BB-Gm (∆*dksA*/Ø) or MRB46 bearing the *dksA*-expressing miniTn*7*BB-Gm[P*dksA-dksA*] (∆*dksA*/*dksA*). Overnight cultures were diluted to an A_600_ of 0.01 and dispensed in three microtiter plates. Plates were sacrificed for measurement at 8 (white bars), 24 (red bars) and 48 hours (blue bars). The plots represent the averages and standard deviations of three independent assays made in sextuplicate

**Supplementary Figure S4. *In vitro* transcription of the P*bifA* and P*aer2* promoters.** Full *in vitro* transcription gels of ppGpp titrations in the presence or absence of DksA on the P*bifA* (a) and P*aer-2* (b) promoters, as shown in Fig. 5c and 5d. Images of transcript levels are as detected by X-ray films, with a longer exposure time of the P*bifA* gel as compared to P*aer2*.

**TABLES**

**Supplementary Table S1. Bacterial strains, plasmids and oligonucleotides used in this work.** Underlined bases indicate oligonucleotide positions that differ from the corresponding templates.^1^References listed in Supplementary Materials Reference list.

**Bacterial strain Genotype/phenotype Reference^1^/source**

***E. coli***

DH5α φ80d*lacZ*∆M15 ∆(*lacZYA-argF*)U169 *recA*1 *endA*1 *hsdR*17 (r_k_^-^ m_k_^+^) *supE*44 *thi*-1 *gyrA* *relA*1 1

***P. putida***

KT2440 mt-2 *hsdR*1 (r^-^ m^+^) 2

KT2440 *fliA*::*aphA-3* KT2440 *fliA*::Km. Km^r^ 3

KT2440-Tel KT2440 tagged with miniTn*5*-Tel. Tel^r^ 4

KT2440::miniTn*7*BB-Gm KT2440 miniTn*7*BB*-*Gm::*glmS* Gm^r^ This work

KT2440::TpMRB58 KT2440 miniTn*7*BB-Gm[P*dksA-dksA*]::*glmS* Gm^r^ This work

KT2442 mt-2 *hsdR*1 (r^-^ m^+^)Rif^r^ 2

MRB1 KT2442 miniTn*5*-Km::*lapG* Rif^r^ Km^r^ 5

MRB46 KT2440 ∆*dksA* This work

MRB46::miniTn*7*BB-Gm MRB46 miniTn*7*BB*-*Gm::*glmS* Gm^r^ This work

MRB46::TpMRB58 MRB46 miniTn*7*BB-Gm[P*dksA-dksA*]::*glmS* Gm^r^ This work

MRB53 KT2440-Tel ∆*dksA* ∆*relA*::Km ∆*spoT*::Gm. Tel^r^ Km^r^ Gm^r^ This work

PP1437 KT2440-Tel ∆*relA*::Km. Tel^r^ Km^r^ This work

PP1922 KT2440-Tel ∆*relA*::Km ∆*spoT*::Gm. Tel^r^ Km^r^ Gm^r^ This work

**Plasmid Genotype/phenotype Reference^1^/source**

pALS13 pKK223-3-derived plasmid producing RelA^∆456-743^ from the P*tac* promoter. Ap^r^ 6

pALS14 pKK223-3-derived plasmid producing RelA^∆332-743^ from the P*tac* promoter. Ap^r^ 6

pBBR1-MCS4 Broad host-range cloning vector. Ap^r^, Mob^+^ 7

pENTR™/D-TOPO® Vector for directional TOPO® cloning. Km^r^ Thermo Fisher Scientific

pEX18-Tc Gene replacement vector with MCS from pUC18. Tc^r^ Sac^s^ Mob^+^ 8

pMPO284 pPS854-derived vector containing pUTminiTn*5*Km Km^r^ gene flanked by the FRT sites, Ap^r^ Km^r^ 9

pMRB1 pBBR1-MCS4-derived broad host-range *gfp*mut3::*lac*Z transcriptional fusion vector. Ap^r^ 9

pMRB2 pMRB1-derived vector containing the Gateway conversion cassette *attR*2-*ccdB*-Cm^r^-*attR*1. Ap^r^ Cm^r^ 9

pMRB3 pMRB1 -derived vector containing the Gateway conversion cassette *attR*1-Cm^r^-*ccdB*-*attR*2. Ap^r^ Cm^r^ 9

pMRB33 pEX18-Tc bearing *dksA* upstream and downstream flanking regions. Tc^r^ Sac^s^ Mob^+^ This work

pMRB38 pMRB33 with Km^r^ gene flanked by FRT sites cloned between *dksA* flanking regions. Tc^r^ Km^r^ Sac^s^ Mob^+^ This work

pMRB58 pUC18Sfi-based delivery plasmid for the minitransposon miniTn*7*BB-Gm[P*dksA-dksA*]. Ap^r^ Gm^r^ 5

pMRB66 pMRB3-derived vector containing a P*lapBC-gfp*mut3-*lac*Z transcriptional fusion. Ap^r^ Mob^+^  This work

pMRB67 pMRB2-derived vector containing a P*lapA-gfp*mut3-*lac*Z transcriptional fusion. Ap^r^ Mob^+^  This work

pMRB68 pMRB3-derived vector containing a P*bifA-gfp*mut3-*lac*Z transcriptional fusion. Ap^r^ Mob^+^  9

pMRB97 pPS854-derived plasmid bearing a Str^r^ Spec^r^ gene between the FRT sites. Ap^r^ Str^r^ Spec^r^ This work

pMRB98 pMRB33 with Str^r^ Spec^r^ gene flanked by FRT sites cloned between *dksA* flanking regions. Tc^r^ Str^r^ Spec^r^ Sac^s^ Mob^+^ This work

pMRB120 pSB1K3 derivative bearing the *nahR*-P*sal* expression cassette. Km^r^ Laura Claret, unpublished

pMRB153 pBBR1-MCS4 derivative bearing a truncated *relA* ORF encoding the RelA^∆456-743^ derivative. Ap^r^ Mob^+^ This work

pMRB154 pBBR1-MCS4 derivative bearing a truncated *relA* ORF encoding the RelA^∆332-743^derivative. Ap^r^ Mob^+^ This work

pMRB160 pBBR1-MCS4 derivative producing RelA^∆456-743^ from the *nahR-*P*sal* expression system. Ap^r^ Mob^+^ This work

pMRB162 pBBR1-MCS4 derivative producing RelA^∆332-743^ from the *nahR-*P*sal* expression system. Ap^r^ Mob^+^ This work

pMRB240 pMRB2-derived vector containing a P*lapE-gfp*mut3-*lac*Z transcriptional fusion. Ap^r^ Mob^+^  This work

pMRB241 pMRB2-derived vector containing a P*lapGD-gfp*mut3-*lac*Z transcriptional fusion. Ap^r^ Mob^+^  This work

pPS854 pUC1819-derived vector containing two FRT sites. Ap^r^ 8

pRK2013 Helper plasmid for triparental mating. ColE1 replicon. Km^r^ 10

pSB1K3 High copy-number cloning vector. Km^r^ 11

pTE103 *In vitro* transcription template plasmid. Ap^r^ 12

pTNS2 R6K replicon-based helper plasmid expressing the Tn*7* transposase. Ap^r^ Mob^+^ 13

pUC18Sfi-miniTn*7*BB-Gm pUC18Sfi-based delivery plasmid for the synthetic minitransposon miniTn7BB-Gm. Ap^r^ Gm^r^ 9

pUTminiTn*5*-Sm/Sp Delivery plasmid for minitransposon miniTn*5*-Sm/Sp. Ap^r^ Str^r^ Spec^r^ Mob^+^ 14

pVI681 Suicide plasmid bearing the gene deletion/replacement allele ∆*relA*::Km. Ap^r^ Km^r^ 4

pVI682 Suicide plasmid bearing the gene deletion/replacement allele ∆*spoT*::Gm. Ap^r^ Gm^r^ 4

pVI1011 pTE103-based *in vitro* transcription template plasmid bearing the P*aer2* promoter. Ap^r^ 15

pVI2407 pTE103-based *in vitro* transcription template plasmid bearing the P*bifA* promoter. Ap^r^ This work

**Oligonucleotide Sequence (5’ to 3’)**

dksAFdwstm ATTAGGATCCATGATCTTTCTTCCACCTGAACG

dksAFupstm ATATGAATTCTCCTTGTCCCAGTAGCGCTC

dksARdwstm ATATAAGCTTCAGTGGCGGCGAACGGTAC

dksARupstm ATATGGATCCACATGAATAGACCGCCTCTCAC

0A4_fwd CACCGCGGGTCTTGTAGGTGTCAT

0A4_rev CGCCTACTACATCGACTACCG

bifA-fwd TACCTGGTGTACCAGCCACA

bifA-rev TTTCGGTGACTTCCAGTTCC

**REFERENCES**

1. Hanahan, D. Studies on transformation of *Escherichia coli* with plasmids. *J. Mol. Biol.* **166**, 557-580 (1983).
2. Franklin, F. C., Bagdasarian, M., Bagdasarian, M. M. & Timmis, K. N. Molecular and functional analysis of the TOL plasmid pWWO from *Pseudomonas putida* and cloning of genes for the entire regulated aromatic ring meta cleavage pathway. *Proc. Natl. Acad. Sci. USA* **78**, 7458-7462 (1981).
3. Rodríguez-Herva, J. J. *et al*. Physiological and transcriptomic characterization of a *fliA* mutant of *Pseudomonas* *putida* KT2440. *Environ. Microbiol. Rep.* **2**, 373-380 (2010).
4. Sze, C. C., Bernardo, L. M. D. & Shingler, V. Integration of global regulation of two aromatic-response σ^54^-dependent systems: a common phenotype by different mechanism. *J. Bacteriol.* **184**, 760-770 (2002).
5. López-Sánchez, A., Jiménez-Fernández, A., Calero, P., Gallego, L. D. & Govantes, F. New methods for the isolation and characterization of biofilm-persistent mutants in *Pseudomonas putida*. *Environ. Microbiol. Rep.* **5**, 679–685 (2013).
6. Svitil, A. L., Cashel, M. & Zyskind, J. W. Guanosine tetraphosphate inhibits protein synthesis *in vivo*. A possible protective mechanism for starvation stress in *Escherichia coli*. *J. Biol. Chem.* **268**, 2307-2311 (1993).
7. Kovach, M. E. *et al.* Four new derivatives of the broad-host-range cloning vector pBBR1MCS, carrying different antibiotic-resistance cassettes. *Gene* **166**, 175-176 (1995).
8. Hoang, T. T., Karkhoff-Schweizer, R. R., Kutchma, A. J. & Schweizer, H. P. A broad-host-range Flp-FRT recombination system for site-specific excision of chromosomally located DNA sequences: application for isolation of unmarked *Pseudomonas aeruginosa* mutants. *Gene* **212**, 77-86 (1998).
9. Jiménez-Fernández, A., López-Sánchez, A., Calero, P. & Govantes, F. The c-di-GMP phosphodiesterase BifA regulates biofilm development in *Pseudomonas putida*. *Environ. Microbiol. Rep.* **7**, 78-84 (2015).
10. Figurski, D. H. & Helinski, D. R. Replication of an origin-containing derivative of plasmid RK2 dependent on a plasmid function provided in *trans*. *Proc. Natl. Acad. Sci. USA* **76**, 1648-1652 (1979).
11. Shetty, R. P., Endy, D. & Knight, T. F., Jr. Engineering BioBrick vectors from BioBrick parts. *J. Biol. Eng.* **2**, 5 (2008).
12. Elliott, T. & Geiduschek, E. P. Defining a bacteriophage T4 late promoter: absence of a ‘−35’ region. *Cell* **36**, 211-219 (1984).
13. Choi, K. H. *et al*. A Tn*7*-based broad-range bacterial cloning and expression system. *Nat. Methods* **2**, 443-448 (2005).
14. de Lorenzo, V., Herrero, M., Jakubzik, U. & Timmis, K. N. Mini-Tn*5* transposon derivatives for insertion mutagenesis, promoter probing, and chromosomal insertion of cloned DNA in Gram-negative eubacteria. *J. Bacteriol.* **172**, 6568-6572 (1990).
15. Österberg, S., Skärfstad, E. & Shingler, V. The σ-factor FliA, ppGpp and DksA coordinate transcriptional control of the *aer2* gene of *Pseudomonas putida*. *Environ. Microbiol.* **12**, 1439-1451 (2010).
